# Intraductal Carcinoma of the Prostate versus Simulants: A Differential Diagnosis Growing in Clinical Impact

**DOI:** 10.3390/cancers16061097

**Published:** 2024-03-08

**Authors:** Steven Christopher Smith, Sara E. Wobker

**Affiliations:** 1 Departments of Pathology and Division of Urology, Department of Surgery, VCU School of Medicine, VCU Massey Comprehensive Cancer Center, and Richmond Veterans Affairs Medical Center, Richmond, VA 23298, USA; 2 Department of Pathology and Laboratory Medicine, University of North Carolina, Chapel Hill, NC 27599, USA; sara_wobker@med.unc.edu

**Keywords:** prostate cancer, gleason grading, grade groups, cribriform pattern, intraductal carcinoma of the prostate

## Abstract

**Simple Summary:**

Though intraductal carcinoma of the prostate has been observed and recognized for years, more recently this process has emerged, to become simultaneously both a consensus, major prognostic factor and controversial parameter as relates to potential impact on the histologic grading in prostate cancer. Due to the increasing incorporation of intraductal carcinoma into risk stratification approaches and management considerations in this disease, the differential diagnoses of this lesion have become of increasing importance. Herein, we review the background of recognition of and evolution of the criteria for intraductal carcinoma, emphasizing its differential diagnoses, both neoplastic and non-neoplastic. We furthermore recommend useful immunohistochemical markers to help correctly recognize and diagnose this key process.

**Abstract:**

Despite its first recognition even longer ago, in the past nearly 20 years, intraductal carcinoma of the prostate has become a standard histopathologic reporting parameter conveying a strong negative prognostic factor for prostatic adenocarcinoma. When seen at biopsy, intraductal carcinoma of the prostate is associated with risk for aggressive prostatectomy outcomes, including frequently high-grade, high-stage, high-volume disease, with increased risk for recurrence and progression. Multiple organizations, including the uropathology subspecialty societies to the World Health Organization, recognize and recommend reporting the presence of intraductal carcinoma, whether sampled in “pure” form or present with concomitant invasive adenocarcinoma. Moreover, emerging scholarship relates intraductal carcinoma to higher prevalence of homologous recombination repair deficiency mutations in prostatic adenocarcinoma, whether somatic or germline, which serve as indications for approved targeted therapies. Taken together, this is a diagnosis for the histopathologist not to miss. In view of these elevated stakes and the opportunity to further precision medicine, this review details neoplastic and non-neoplastic simulants in the differential diagnosis of intraductal carcinoma of the prostate.

## 1. Introduction

The phenomenon now known as *intraductal carcinoma of the prostate* has likely been observed in a subset of cases for as long as the diagnostic histopathology of prostatic adenocarcinoma has been reviewed by surgical pathologists. It is generally acknowledged that one of the earliest descriptions of cancerization of pre-existing ducts of the prostate by prostatic adenocarcinoma was by Kovi et al. in *Cancer* in 1985 [1]. This remarkable report built on relatively recent descriptions of how cancer cells can invade ducts in a retrograde fashion from their peripheral stromal interface, to characterize the mechanism of ductal spread in prostate cancer. Even more remarkably, in an era before immunohistochemistry, by light microscopic criteria and histochemical stains, the authors not only described this phenomenon but attempted to delineate its relative prevalence (which they estimated at nearly half of cases, whether of prostatectomies or transurethral resections). It remains striking that they even identified that “ductal spread” was statistically significantly proportional to the “local extent” (which they defined as cancer volume estimated by proportion of resected tissue fragments involved by cancer). They observed its association with Gleason grade as well, though in multivariate analysis, local extent appeared more strongly correlated to grade (though extent and grade were also both tight covariates). They further characterized three patterns of duct involvement, by retrograde “microinvasion” through the epithelial basement membrane, “permeation” where basement membrane integrity was somewhat preserved, and “extension in continuity”, where carcinoma was seen proliferating within and between the luminal surface cells and basal cells. While the described phenomena likely included what we would now designate as high-grade prostatic intraepithelial neoplasia (HGPIN), atypical intraductal proliferations suspicious for intraductal carcinoma, and intraductal carcinoma itself, this scholarship was remarkable in its anticipation of the future direction of the field. 

Additional descriptions by McNeal followed one year later, where cribriform adenocarcinoma was described as “in most cases…predominantly intraductal” and associated with a higher proportion of Gleason pattern 4 and 5 areas [2]. This report further included description of cribriform carcinoma as frequently “representing intraductal carcinoma”. The same author further studied “duct lumen-spanning” lesions, separating them from “dysplasia”, as HGPIN was termed at the time, in an additional report in 1996 [3]. In this later report, for the first time intraductal carcinoma (as well as pattern 4 and 5 cancer and perineural spread) was related to post-prostatectomy biochemical recurrence, even beyond prostatectomy findings such as extracapsular extension and surgical margins. Quite presciently, McNeal and Yemoto postulated that intraductal carcinoma may have a “unique biologic significance” reflective of capacity for extensive spread within ducts, among other parameters of aggression [3]. The phenomenon of intraductal carcinoma continued to be studied episodically under various names and from varied perspectives through the turn of the millennium [4,5,6,7,8]. 

A rapid growth in interest in intraductal carcinoma of the prostate was triggered by an influential biopsy-based study published in 2006 by Guo and Epstein [9]. In this study, the diagnostic histopathologic features, and the clinical outcomes of a cohort of 27 *pure* intraductal carcinomas detected at prostatic needle core biopsy (and proven as intraductal through the use of basal cell marker immunohistochemistry) were examined, and a remarkably useful set of criteria were proposed. Specifically, they defined these lesions as a proliferation of malignant epithelial cells, filling large acini and ducts with preserved basal cells, showing either a solid or dense cribriform pattern, or, absent these, a loose cribriform or micropapillary pattern if present with either marked nuclear atypia (nuclear size > 6-fold the size of a normal nucleus) or with comedonecrosis. When available prostatectomy outcomes were assessed for such lesions, Gleason score 8 or 9 carcinoma was always present, as was, in the majority, extraprostatic extension. Bone metastasis was identified among three of sixteen cases which were not treated with resection. The authors interpreted these findings as documenting an important new biopsy diagnosis, tightly associated with clinically significant, often aggressive, disease. Moreover, they recommended that when encountered prospectively in the biopsy setting, definitive treatment should be considered even without documentation of invasive carcinoma. 

Subsequent scholarship, including a larger cohort focusing on prostatectomy outcomes [10], supported this recommendation. Moreover, when in the presence of invasive carcinoma, numerous reports and even systematic reviews have subsequently documented that intraductal carcinoma is a significant negative prognostic factor [11,12,13,14,15,16,17]. Based on this scholarship, intraductal carcinoma has become a recommended reporting parameter, since the 2014 International Society of Urological Pathology (ISUP) Consensus [18], and recognized for the first time as an entity by the World Health Organization in 2016 [19], a category maintained in the current 5th Edition Classification [20]. Subsequent ISUP [21] and Genitourinary Pathology Society (GUPS) [22] recommendations have further supported reporting intraductal carcinoma, whether encountered in “pure” form without invasive cancer, a scenario where neither group recommends assigning it a grade. When encountered with concomitant invasive carcinoma, the groups differ in recommendation [23,24] over whether to include the non-invasive pattern seen within the ducts in grade assignment for the invasive carcinoma. This difference in practice reflects significantly different perspectives on the appropriateness of this non-invasive lesion contributing to grade assignment (for example [25]) and significant interobserver practice differences in criteria, grading, and immunostain utilization that we [26] and others [27] have observed. We posit that prospective scholarship should be the solution to these conundrums. 

More relevant to the differential diagnosis of intraductal carcinoma, the criteria for diagnosis of intraductal carcinoma have evolved significantly if marginally from those described in 2006 [9], which had been echoed in the 2016 WHO Classification [19]. Subsequent criteria discussed in an influential review addressed the definition of the “density” of the intraductal proliferation, discussing a 70% cutoff for the ratio of intraductal neoplastic cells versus empty space [28], while most recently the GUPS 2019 White Paper espoused a somewhat looser criterion of at least a 50% ratio of epithelium to glandular spaces to consider an intraductal proliferation “dense”. GUPS recommended an overall criterion stating “dense cribriform glands and/or solid nests and/or marked pleomorphism/necrosis. Dense cribriform glands are defined as 50% of the gland composed of epithelium relative to luminal spaces” [22]. This somewhat lower threshold for diagnosis nonetheless came with the recommendation to regard cases hovering at around a 50% ratio of epithelial proliferation to luminal white space as “atypical intraductal proliferation” and suspicious for but not diagnostic of intraductal carcinoma. Lastly, the most recent WHO Classification parses the diagnostic criteria for intraductal carcinoma into *essential* and *desirable* features, as per the edition’s convention across entities [20]. The chapter on intraductal carcinoma specifies the essential criteria of “expansile epithelial proliferation in the pre-existing duct-acinar system; lumen-spanning solid, cribriform, and/or comedo patterns; loose cribriform or micropapillary patterns with enlarged pleomorphic nuclei; residual basal cells” and the desirable criterion of immunohistochemistry demonstrating at least partial basal cell retention. Indeed, the retention of basal cells can be variable or focal in different examples of intraductal proliferations [29]. In any case, the issue of atypical intraductal proliferations falling short of criteria for intraductal carcinoma concerns one of the important differential diagnoses discussed, as discussed below.

One additional area where data are lacking and criteria are somewhat limited concerns the marked propensity of the ductal subtype of prostatic adenocarcinoma to spread intraductally [30]. Ductal adenocarcinoma, which is defined histomorphologically, perhaps most specifically, cytologically, exhibits tall columnar cells with a pseudostratified epithelial arrangement, elongate nuclei with prominent nucleoli and frequent mitosis, and true, complex papillary and cribriform architecture. Specifically, in many areas of sections of classic ductal adenocarcinomas, especially those arising in the gland centrally in a periurethral distribution involving ducts, basal cells are frequently detectable by immunohistochemistry for markers such as 34βE12, a phenomenon which may simulate high grade prostatic intraepithelial neoplasia in biopsies [31]. This phenomenon is well characterized in the literature regarding prostatic ductal adenocarcinoma itself, case series of which contend that >90% of well characterized prostatectomy samples of ductal adenocarcinoma exhibit intraductal growth patterns as a component [32]. That said, surprisingly, despite the current emphasis on intraductal carcinoma, recent society consensus and WHO classifications have not comprehensively addressed the conceptual and verbal conundrum of “intraductal ductal”. Perhaps most usefully, the 2019 GUPS White paper, noted the existence of cases of intraductal carcinoma with this pattern, recommending the term intraductal carcinoma of the prostate “with ductal morphology” for when such a scenario is encountered [22], and acknowledging the limited data on the relative prevalence of this finding among biopsy cases of intraductal carcinoma. 

As bears on the stakes of the differential diagnosis of intraductal carcinoma of the prostate, a brief consideration of the most direct clinical implications of this diagnosis is warranted. Given the very strong association of intraductal carcinoma with aggressive cancer outcomes, it is somewhat unexpected that it is not a direct contributor to the initial risk stratification or treatment pathway guidelines of the United States National Comprehensive Cancer Center Network (NCCN) [33] nor American Urological Association (AUA) [34]. At most, the NCCN mentions that nomograms have been published using intraductal carcinoma and other features that outperform the NCCCN risk groups; the AUA guidelines simply state intraductal carcinoma is a feature for discussion when counseling a patient [34]. In contrast, the current intersociety guidelines led by the European Association of Urology (EAU) [35], building on a recent consensus [36] on criteria for deferred active treatment (i.e., active surveillance and related approaches), *explicitly exclude patients with intraductal carcinoma from active surveillance*. In contrast, the most explicit recommendation stemming from a diagnosis of intraductal carcinoma in the NCCCN guidelines is for consideration of germline genetic testing for patients [33], echoed also by the AUA for intermediate risk patients [34]. 

A developing body of work, albeit one with several contradictory findings, has suggested that intraductal carcinoma is enriched for increased prevalence of pathogenic variants in DNA repair pathways, especially homologous recombination repair (HRR) deficiency. Genes in this pathway include (at least) *BRCA1*, *BRCA2*, *ATM*, *CHEK2*, *PALB2*, *FANCA*, *RAD51B*, and *BRIP*. At present, it is unclear whether the mutations seen are more frequently germline or somatic, and whether intraductal carcinoma is a specific marker of HRR deficiency or simply prevalent among advanced/aggressive cancers where HRR deficiency also is prevalent (and studied for treatment purposes) [37,38,39,40,41,42,43]. In any case, in advanced or metastatic prostate cancers, the prevalence of germline or somatic HRR mutations has been estimated at 20% [44] and may approach 30% in some cohorts [45] depending on selection. From the standpoint of intraductal carcinoma differential diagnosis, suffice it to say that if diagnosed, the treating oncology team may consider the patient particularly appropriate for either germline or somatic comprehensive genomic profiling. 

## 2. Neoplastic Simulants of Intraductal Carcinoma

### 2.1. High-Grade Prostatic Intraepithelial Neoplasia

**Figure 1:**
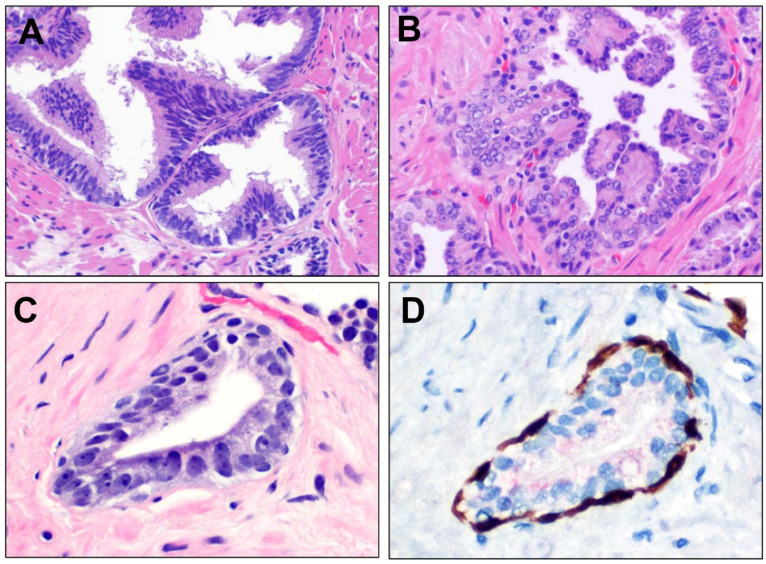
Patterns of high-grade prostatic intraepithelial neoplasia (HGPIN). (**A**) Classic examples of HGPIN demonstrate a tufted appearance, with areas of focal accentuation of pseudostratified columnar luminal epithelial cells showing atypia in the form of nucleomegaly, nuclear hyperchromasia, and nucleoli (200×). (**B**) An infrequent pattern of HGPIN is that of inverted HGPIN, where an acinus is involved by a neoplastic epithelium with a tufted or micropapillary configuration, but where the nucleoli are located adluminally, lifted off the basement membrane, rather than more basally (200×). (**C**) Flat HGPIN is a less prevalent pattern where a flat, lower cuboidal atypical epithelium is present, rather than the usual tall, columnar cells, with tufted or micropapillary architecture. This pattern is principally notable due to its potential simulation of invasive carcinoma (400×). (**D**) As is the case for all HGPIN variants, in flat HGPIN, positive staining for basal cells (p63 nuclear/HMWCK cytoplasm, brown chromogen) and variable overexpression of AMACR (red chromogen) can readily be demonstrated by multiplex immunohistochemistry (400×).

High-grade Prostatic Intraepithelial Neoplasia (HGPIN) is regarded as a non-obligate, non-invasive precursor of acinar subtype prostatic adenocarcinoma [46,47]. HGPIN is characterized by intra-acinar proliferation of neoplastic but pre-malignant luminal cells exhibiting nuclear atypia, including nucleomegaly and nucleolar prominence, but is nonetheless bounded by basal cells. Interobserver reproducibility studies have suggested that “for a diagnosis of HGPIN, atypical glands should be evident at scanning magnification, typically as architecturally complex glands with hyperchromasia that stand out from the benign glands. Confirmation of HGPIN is made by examining the glands at 20× magnification to confirm the presence of conspicuous nucleoli in the luminal cells” [48]. HGPIN may variably demonstrate tufted, micropapillary, and flat patterns, as well as an unusual “inverted” pattern (Figure 1). 

**Figure 2:**
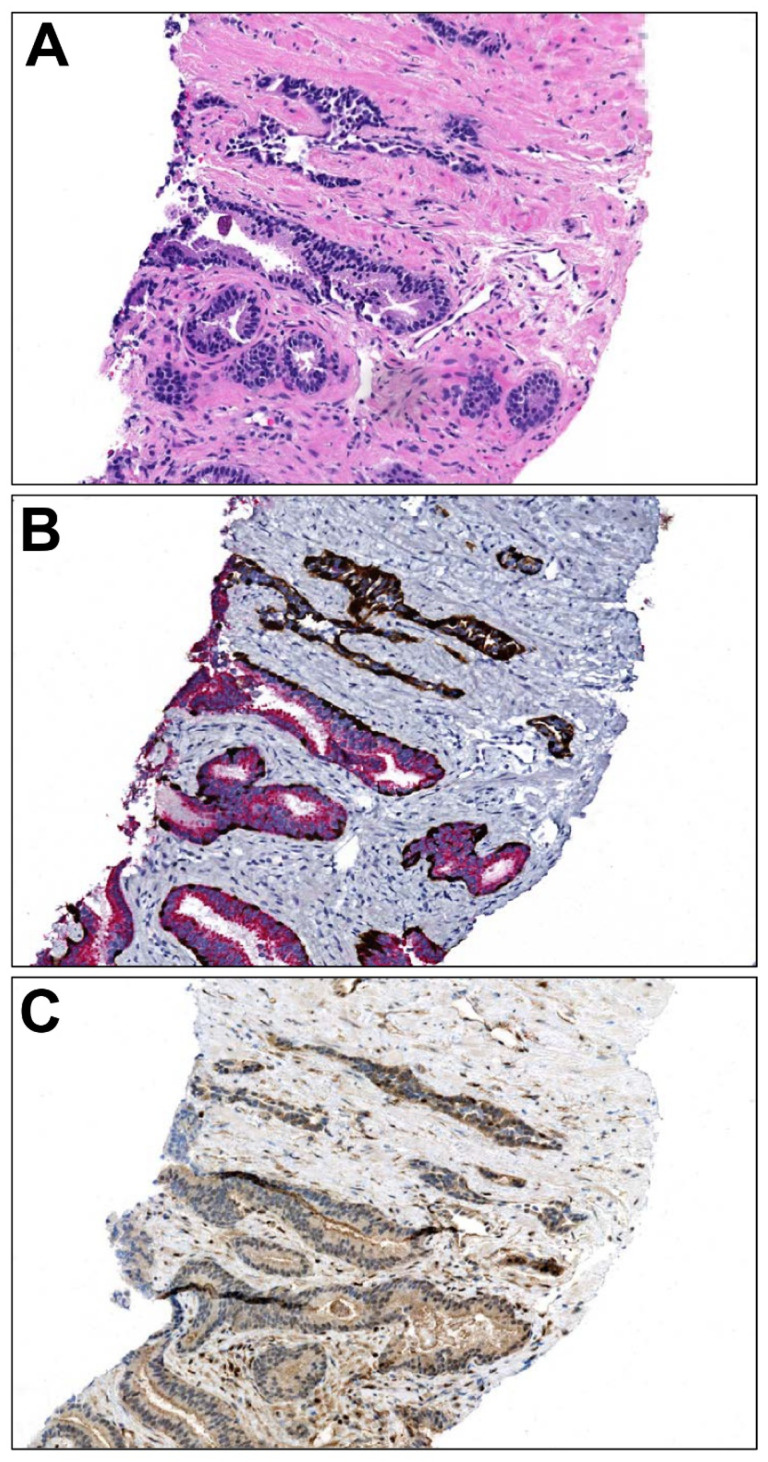
Conventional HGPIN immunophenotype. (**A**) A focus of HGPIN demonstrates at low power larger nuclei and hyperchromatic amphophilic cytoplasm (lower half field) as compared to atrophic ducts and acini (upper half of the field) (100×). (**B**) Retained expression of p63 nuclear/HMWCK cytoplasmic positive peripheral basal cells (brown chromogen) is characteristic, as frequently is overexpression of AMACR (red chromogen) (100×). (**C**) Recent studies have established that HGPIN retains expression of PTEN (brown chromogen, cytoplasmic and membranous expression), which is distinctive in the differential diagnosis versus intraductal carcinoma (100×).

Of note, the previously described “cribriform” pattern of HGPIN is no longer diagnosed, given that it represents either an atypical intraductal proliferation that is at least suspicious for intraductal carcinoma, or, in the presence of sufficient atypia, intraductal carcinoma itself. Because HGPIN is itself benign, and, when encountered at biopsy, only associated with a very mildly elevated risk of adenocarcinoma on subsequent biopsy [49,50,51], its distinction from an aggressive pattern of disease such as intraductal carcinoma is of paramount importance. Qualitative features favoring HGPIN and against consideration of intraductal carcinoma include the smaller size of the focus involved (similar to adjacent/unequivocally benign acini), smooth peripheral contours, lack of expansion and or branching of the acinus by the epithelial proliferation, lower degree of nuclear atypia, lack of mitosis, lack of comedonecrosis, lack of associated invasive adenocarcinoma, and more extensive preservation of basal cells. Immunohistochemistry also has a role in this context, beyond the ability to prove preservation of basal cells; isolated HGPIN expressing ERG is distinctly unusual [52], while, perhaps more usefully, retention of PTEN staining is nearly always present [52,53,54,55] (Figure 2). 

### 2.2. Atypical Intraductal Proliferation

**Figure 3:**
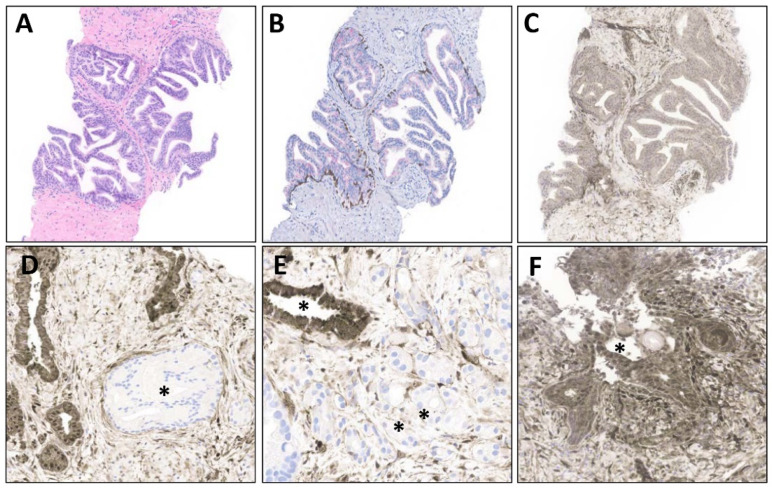
The role of PTEN in the differential diagnosis of florid HGPIN and atypical intraductal proliferations (AIPs). (**A**) A focus showing three tangentially sectioned acini of florid HGPIN raises consideration of atypical intraductal proliferations due to the density and complexity of the epithelial proliferations (40×). (**B**) The intraductal nature of these three HGPIN acini is confirmed with p63 nuclear/HMWCK cytoplasmic (brown chromogen) peripheral basal cell staining, with mild overexpression of AMACR (red chromogen) (40×). (**C**) This focus demonstrates retained PTEN expression (brown chromogen), favoring interpretation as florid HGPIN rather than AIP or intraductal carcinoma (40×). (**D**) In contrast, this dense, lumen spanning, but unusually small intraductal focus (asterisked) shows loss of expression of PTEN (brown chromogen) in the lesional cells, with internal positive control benign ductal cells (left) strongly positive, favoring interpretation as an AIP (200×). (**E**) An anatomically adjacent core biopsy to the site of (**D**) demonstrates invasive carcinoma, also PTEN negative (double asterisk, right lower field), as compared to strongly retained expression in benign adjacent ducts (single asterisk). Carcinoma, particularly higher grades, shows frequent loss of PTEN (200×). (**F**) PTEN is also strongly retained in this reactive/metaplastic ductal focus (asterisk), where inflammation might simulate necrosis and induce reactive nuclear atypia (200×).

Several names have been used to refer to intraductal epithelial proliferations that are worrisome, with findings beyond that allowable in HGPIN, but where diagnostic criteria for intraductal carcinoma are not met. These include *atypical cribriform proliferation*, *atypical intraductal cribriform proliferation*, *low-grade intraductal carcinoma*, and even *atypical proliferation suspicious for intraductal carcinoma*, as reviewed recently in the 2019 GUPS White Paper [22] on grading prostate cancer. In fact, *atypical intraductal proliferation* (AIP) is the recommended term, given that it subtends the full morphologic spectrum of patterns, beyond the common cribriform pattern; this term is noted to be preferred in the 5th Edition WHO classification [20]. While an AIP may be diagnosed in principle based on a suspicious intraductal focus showing deficiency of any of the required criteria for diagnosis of intraductal carcinoma [29,54,56,57,58,59], it is thought that most such cases demonstrate a pattern of intraluminal cribriform growth that is not solid or dense enough for a definitive designation [56]. In any case, these lesions show features that closely recapitulate intraductal carcinoma [54,58,60,61], such that authors recommend immediate rebiopsy [54,58]. Given that the term is still less widely understood by clinicians, when used, an explanation of the “suspicious for but not definitive for” intraductal carcinoma is recommended in a comment, and immediate rebiopsy and/or imaging guided rebiopsy recommended from a management standpoint. While by definition a combination of routine H&E and basal cell marker immunostains are sufficient for diagnosis, immunohistochemistry for PTEN also appears to be useful, with regards to the differential diagnosis with HGPIN [47,52,54], given its retained expression in HGPIN and frequent loss in both AIP and intraductal carcinoma [54,58] (Figure 3). Table 1 briefly summarizes the immunohistochemical biomarkers used in this differential diagnosis, alongside those relevant for the foregoing and subsequent entities described. 

### 2.3. High-Grade Invasive Adenocarcinoma Patterns

High-grade patterns of prostatic adenocarcinoma may simulate intraductal carcinoma due to the overlapping morphologies present, including invasive solid, cribriform, and comedonecrosis patterns. Firstly, significant recent scholarship suggests that comedocarcinoma is quite frequently but not exclusively a pattern of intraductal carcinoma [67,68], a scenario where basal cell marker immunohistochemistry frequently must be performed for definitive classification as intraductal or invasive. Certainly, solid Gleason pattern 5 invasive adenocarcinoma, if showing rounded contours, may also raise consideration of the solid pattern of intraductal carcinoma. Perhaps the most challenging differential diagnosis with intraductal carcinoma is invasive cribriform adenocarcinoma. Cribriform carcinoma has been recognized as a pattern of invasive prostatic adenocarcinoma, going back to Gleason’s original description and modifications [69,70,71,72], while the 2005 ISUP consensus largely reallocated this pattern to Gleason pattern 4 (at least if large or irregularly circumscribed) [73]. While earlier scholarship had identified the potential for cribriform growth to signal aggressive behavior in prostate cancer [2,3]. However, more recent scholarship from cases diagnosed post-ISUP 2005 provided strong evidence that large and small cribriform growth patterns were independently associated with risk of post prostatectomy biochemical failure [74]. In the ensuing several years after this work, a significant number of studies identified cribriform pattern as a key and often independent prognostic factor [16,75,76,77,78,79,80], as reviewed recently [60,81], and further supported by a contemporary systematic review and meta-analysis [82]. Consistent with this experience, both GUPS [22] and ISUP [21] agreed in 2019 that reporting cribriform pattern of growth is requisite at biopsy and prostatectomy, which is echoed by the 5th Edition WHO [20]. Moreover, ISUP has promulgated a standardized definition of the cribriform growth pattern: “a confluent sheet of contiguous malignant epithelial cells with multiple glandular lumina that are easily visible at low power (objective magnification ×10). There should be no intervening stroma or mucin separating individual or fused glandular structures” [83]. 

Of note, only a subset of the studies of cribriform growth pattern have specified or specifically assayed whether the pattern seen and assessed prognostically was intraductal or not. Similarly, neither of the societies’ reporting guidelines, nor the ISUP recommended standardized diagnostic criteria for “cribriform”, specifies whether the pattern seen should be invasive or intraductal for recommended reporting purposes. Certainly, from the standpoint of the most salient difference between GUPS and ISUP 2019 recommendations, this is an important issue if employing the GUPS convention not to grade the non-invasive cribriform component [24]. Fortunately, aside from the question of whichever grading and reporting convention is used, resolution of the differential diagnosis of intraductal carcinoma with a cribriform pattern *versus* invasive cribriform patterns of carcinoma is readily addressed by contemporary basal cell markers or PIN cocktails. 

### 2.4. Cancerization of Prostatic Ducts by Urothelial Carcinoma

**Figure 4:**
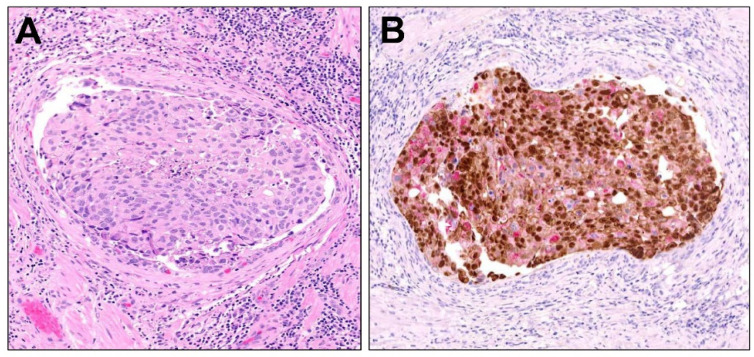
Cancerization of prostatic ducts by urothelial carcinoma. (**A**) In this focus from a transurethral resection of the prostate, urothelial carcinoma in situ has cancerized and expanded a periurethral gland duct. The dense, lumen spanning pattern and nuclear atypia might raise consideration of intraductal carcinoma (200×). (**B**) Multiplex PIN cocktail staining (p63 nuclear/HMWCK cytoplasmic, brown chromogen; AMACR red chromogen) can easily distinguish urothelial carcinoma in situ involving prostatic ducts because of its characteristic p63/HMWCK double positive and AMACR variable expression pattern. This phenotype is distinctive from the p63/HMWCK negative and AMACR positive phenotype of intraductal carcinoma (200×).

Significant recent scholarship has investigated the patterns of disease, differential diagnosis, and staging implications of urothelial carcinoma and its involvement of the prostate. For context, it bears consideration that the staging of urothelial carcinoma regarding its involvement of the prostate gland was changed in the 8th Edition AJCC staging manual [84] to distinguish between direct invasion of urothelial carcinoma through the wall of the bladder into the prostate versus secondary spread into the prostate through extension along the urethra. Specifically, invasion out of the wall of the bladder and then into the prostate is staged as pT4a with respect to the bladder primary, conveying a worse prognosis. When urothelial carcinoma in situ extends along the urethral mucosal surface and into prostatic ducts, it is staged using a separate urethral scheme as either pTis (non-invasive); pT1, invading subepithelial stroma at the urethral surface; or pT2 if invading the deeper prostatic stromal parenchyma. Either way, these patterns of secondary involvement of the prostate through urethral extension are staged and synoptically reported separately as urothelial carcinoma involving the prostate, and each of these stages are prognostically more favorable than direct pT4a from the bladder [85]. Very recent evidence [86] suggests that complete sampling of the prostate in cystoprostatectomy cases may detect significantly more ductal and acinar cancerization by urothelial carcinoma as compared to a more minimalist sampling of the prostate, while incidental insignificant and significant prostatic adenocarcinoma were also more frequently detected. Thus, urothelial carcinoma with in situ cancerization of prostatic ducts may simulate intraductal carcinoma with a solid growth pattern (Figure 4). 

Fortunately, the question of the use of immunohistochemistry for the differential diagnosis of urothelial and prostatic adenocarcinoma has been addressed in detail, including with best practices recommendations promulgated by ISUP [62,63]; urothelial carcinoma frequently expresses p63, HMWCK, CK5/6, GATA3, and CK7, along with Uroplakins, which are all negative in prostatic luminal neoplasms excepting rare prostatic adenocarcinomas with aberrant p63 expression [87,88]. When applied, contemporary prostatic markers such as NKX3.1 and p501S are negative in urothelial carcinoma [89,90]. Specifically, these standard markers remain useful whether considering invasive or intraductal prostatic and urothelial neoplasms. Pertinent to this differential diagnosis discussion is also one caveat, that GATA3 often stains the basal cells of the prostate, especially in the setting of atrophy and post radiation [91,92], while very recent scholarship has indicated D2-40 as a marker of prostatic basal cells that (unlike p63, p40, CK5/6, or HMWCK) is negative in both urothelial carcinoma and intraductal carcinoma of the prostate. This marker may have a role in interpretation of invasiveness in this setting [64]. The stakes of this differential diagnosis are quite significant, especially in the biopsy setting, given the marked differences in the management of urothelial carcinoma versus prostatic adenocarcinoma [93]. It is prudent that any high grade, solid proliferation without evidence of usual acinar prostatic adenocarcinoma should be evaluated with a limited panel of urothelial and prostate markers to confirm the histologic impression of origin. 

### 2.5. Adenoid Cystic (Basal Cell Carcinoma) of the Prostate

**Figure 5:**
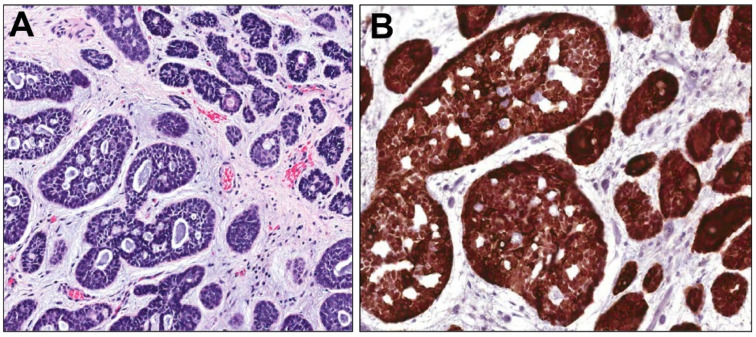
Adenoid Cystic (Basal Cell) Carcinoma of the prostate. (**A**) Adenoid cystic carcinoma of the prostate demonstrates an infiltrative neoplasm with solid (basal cell pattern, upper right) or cribriform (adenoid cystic pattern, lower left) growth, composed of cells with high nucleocytoplasmic ratios. These patterns might engender consideration of solid or cribriform intraductal carcinoma (100×). (**B**) By multiplex PIN cocktail staining, adenoid cystic carcinoma shows diffuse nuclear p63 and cytoplasmic HMWCK (both brown chromogen), while AMACR (red chromogen) is negative (200×).

A potential additional neoplastic simulant of intraductal carcinoma, whether with respect to solid or cribriform growth pattern, is adenoid cystic (basal cell) carcinoma of the prostate gland. This tumor is characterized by variably-sized, infiltrative nests showing either a cribriform (adenoid cystic pattern) or solid (basal cell carcinoma pattern) architecture and composition by small cells with limited cytoplasm and high nuclear to cytoplasmic ratios. Like other basaloid neoplasms, a peripheral palisading of cells at the stromal interface may be apparent. These tumors were recently re-renamed in the 5th Edition WHO classification as adenoid cystic carcinoma, reflecting *MYB::NFIB* fusions identified among a significant subset (especially those with cribriform morphology), as seen in adenoid cystic carcinomas of the salivary glands and other anatomic sites [65,94] (Figure 5). Fortunately, in the differential diagnosis with intraductal carcinoma of the prostate, these tumors express basal cell markers (e.g., p63, HMWCK, p40, especially diffusely at the periphery of the nests, and are negative for luminal cell and neoplastic luminal cell markers such as AMACR and PSA. One immunohistochemical caveat is that loss of expression of PTEN (an aforementioned feature of intraductal carcinoma) has been characterized in these tumors [66]. Given the rarity of adenoid cystic carcinoma of the prostate, treatment guidelines are not well established, though in light of the frequent aggression of these tumors, including extraprostatic extension and propensity for metastasis, at least aggressive local control is indicated [95,96,97]. The importance of this differential diagnosis is highlighted by a recent review of published reported treatments of this entity, which generally document lack of response to treatments directed at conventional prostatic adenocarcinoma, including androgen deprivation therapy, and instead favor aggressive local surgery with consideration of adjuvant radiotherapy [98]. 

## 3. Non-Neoplastic Simulants

### Benign, Metaplastic, and Hyperplastic Processes

Finally, several benign processes, including normal histoanatomic variations, may produce structures, solid and cribriform, that can simulate intraductal carcinoma. In general, across this category, the issue is architectural simulation of intraductal carcinoma, principally benign basal-cell lined processes that simulate the characteristic dense, lumen-spanning features of intraductal carcinoma. Thus, it is the issue of cytologic atypia that is the best distinguishing factor between these processes and intraductal carcinoma. Firstly, particularly in the base of the prostate, the central zone, architecturally complex, even cribriform benign glands may simulate intraductal carcinoma, though overall the maximum atypia expected and density of the proliferation seen is more in the range of HGPIN than necessarily intraductal carcinoma [99]. Additionally, in the region of the verumontanum, architecturally complex seromucinous glands may impart a density and degree of epithelial complexity, again residing in basal cell-lined acini and ducts, that could raise consideration of carcinoma, including intraductal carcinoma, in a limited or needle biopsy sample [100,101]. 

**Figure 6:**
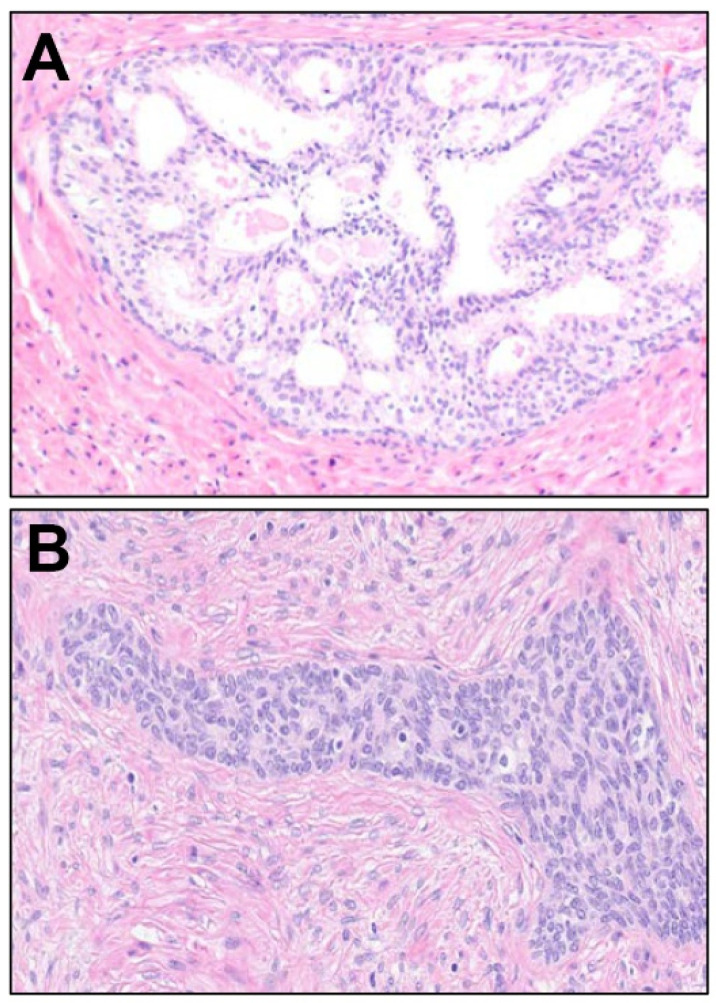
Benign processes that may simulate intraductal carcinoma. (**A**) Clear cell cribriform hyperplasia shows a pattern of exuberant, benign growth demonstrating cribriform, arching, and Roman bridges-like architecture, occurring with some frequency in the base/central zone of the prostate. Fortunately, the degree of nuclear atypia seen in such foci is very mild, most unlike intraductal carcinoma and cribriform invasive carcinoma (200×). (**B**) Basal cell hyperplasia with urothelial metaplasia, frequently present in varying proportions together, may simulate the dense solid pattern of intraductal carcinoma, and may show a branching configuration reminiscent of the branching patters of ducts expanded by intraductal carcinoma. The uniformity of the hyperplastic nuclei, the “streaming” look of spindled cells, and the lack of nuclear atypia beyond grooves, are distinctive from intraductal carcinoma (200×).

Given the often-expanded appearance of the ducts and acini involved by intraductal carcinoma, perhaps some of the most challenging entities in the benign differential diagnosis are hyperplasia, or metaplasia, especially exuberant examples (Figure 6). Clear cell cribriform hyperplasia, as a classic simulant of cribriform Gleason pattern 4 invasive adenocarcinoma, might be considered an even more apt simulant of cribriform intraductal carcinoma, given the presence of basal cells. The lack of admixed invasive adenocarcinoma, lower degree of atypia, and frequently less pronounced AMACR expression are distinguishing *vis a vis* intraductal carcinoma. Basal cell hyperplasia may also show a florid solid or cribriform pattern that might be taken to simulate intraductal carcinoma [97,102]. Moreover, various metaplastic changes, particularly if florid or arising in hyperplastic processes, may also simulate solid or cribriform intraductal carcinoma patterns. These include solid patterns of urothelial or even squamous metaplasia. Certainly, neither of these metaplastic patterns should present the degree of atypia (including nuclear size and nucleolar prominence) generally present in intraductal carcinoma of the prostate. Squamous and urothelial metaplasia both also exhibit a p63/p40 and HMWCK positive immunophenotype, while negative for PSA, AMACR, NKX3.1, and p501S, unlike intraductal carcinoma. Given that intraductal carcinoma is widely regarded as a clinically significant and clinically actionable pattern of carcinoma, the importance of not mislabeling one of these benign processes as intraductal carcinoma is manifest.

## 4. Conclusions

The accurate diagnosis of intraductal carcinoma of the prostate based on its features of lumen-spanning proliferation expanding ducts and acini with atypical cells requires knowledge of its simulants. This review covers the most common pre-neoplastic, malignant, and benign histopathologic mimics of intraductal carcinoma that may introduce difficulty in the diagnosis. The recognition of intraductal carcinoma as a negative prognostic marker has been well-established over the past several decades, leading to development of refined diagnostic criteria and inclusion in reporting schemata. While its diagnostic importance is generally agreed upon, the convergence of accelerated clinical and translational study and widespread clinician awareness [103] of intraductal carcinoma have raised grading related controversy [25] in prostate cancer diagnosis and reporting that is still being argued. While the grading controversy [24] might be subsumed with new histologic stratification systems independent of the Gleason grading heritage and context, at minimum future studies must address the fundamental question of whether biologic and prognostic aspects are defined by the pattern (solid, comedocarcinoma, or cribriform) or the distinctiveness of ductal cancerization reflected in histology. Biomarkers may assist in this regard, especially regarding the emerging conundrum of bona fide, molecularly unique non-invasive precursor-type intraductal carcinomas, unassociated with invasive carcinoma [29,104,105]. Until such a time, we recommend closely following the recommendations of the WHO regarding reporting intraductal carcinoma in all cases, as well as disclosure of whether GUPS or ISUP recommended grading practices are employed when associated with invasive carcinoma. 

## Figures and Tables

**Table 1:** Immunohistochemical (IHC) biomarkers in entities ^1^ in the differential diagnosis of intraductal carcinoma of the prostate.

Entity ^1^	PIN Cocktail ^2^	PTEN IHC	Additional Markers
IDCP [21,22,62]	At least focally present p63/HMWCK positive peripheral basal cells; frequent AMACR overexpression	Loss of expression	Positive: PSA, PSAP, NKX3.1, p501S IHC
HGPIN [53,55,62]	Diffusely retained p63/HMWCK positive peripheral basal cells; frequent AMACR overexpression	Retained membranocytoplasmic expression	Positive: PSA, PSAP, NKX3.1, p501S IHC
AIP [54,55]	At least focally present p63/HMWCK positive peripheral basal cells; frequent AMACR overexpression	Loss of expression	Positive: PSA, PSAP, NKX3.1, p501S IHC
Invasive Pca [62]	Lack of p63/HMWCK positive basal cells; frequent AMACR overexpression	Frequent loss of expression, especially in higher-grade PCa	Positive: PSA, PSAP, NKX3.1, p501S
UC, in prostatic ducts [63,64]	Diffuse expression of p63/HMWCK within the lesional intraductal UC cells	Variably retained or lost expression	Positive: GATA3, p40, CK7, Uroplakins; Negative: D2-40, PSA, NKX3.1, p501S IHC
ACC [65,66]	Diffuse p63 and HMWCK	Frequent loss	*MYB-NFIB* rearrangements in a subset

^1^ IDC,: intaductal carcinoma of the prostate; HGPIN, high-grade prostatic intraepithelial neoplasia; AIP, atypical intraductal proliferation; PCa, prostatic adenocarcinoma; UC, urothelial carcinoma; ACC, adenoid cystic (basal cell) carcinoma of the prostate. ^2^ PIN Cocktail, standard triple multiplex immunohistochemistry for prostatic adenocarcinoma workup, including p63 (nuclear basal cell marker), HMWCK (34βE12) and AMACR (alpha methyl acyl coenzyme A racemase, p504S).
